# Supplementary figures and images for: Ontogenetic, dietary, and environmental shifts in Mesosauridae
Source: PeerJ. 2022 Sep 16;10:e13866. doi: 10.7717/peerj.13866 (PMC9484468; doi:10.7717/peerj.13866)

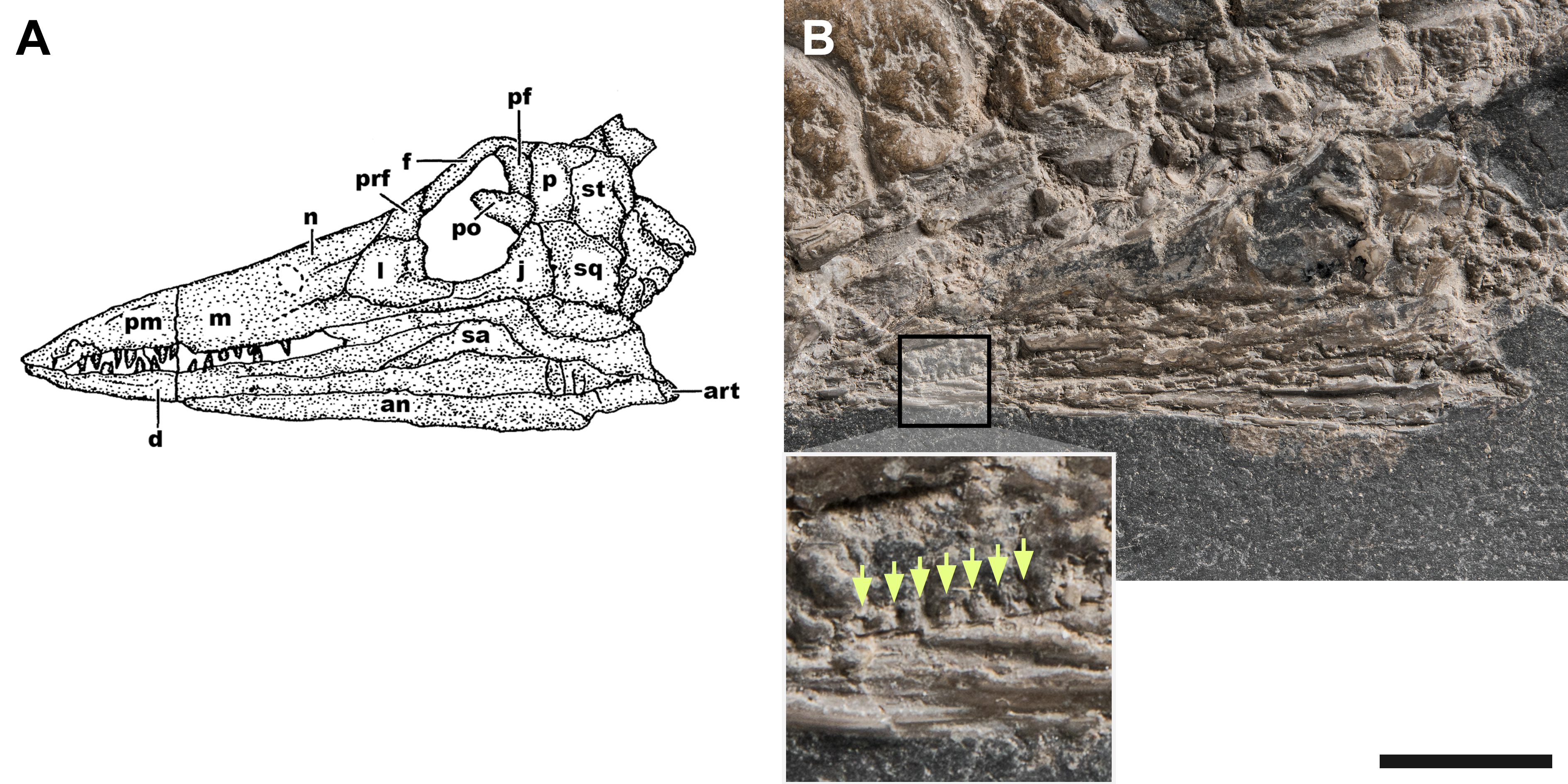

Supplement: Figure S2 — A. Interpretative drawing from Rossmann (2002) showing short conical teeth. B. High-resolution close-up photograph of the skull, showing that the teeth are in fact broken tooth sockets from the dentary (indicated by green arrows). [file peerj-10-13866-s002.png]

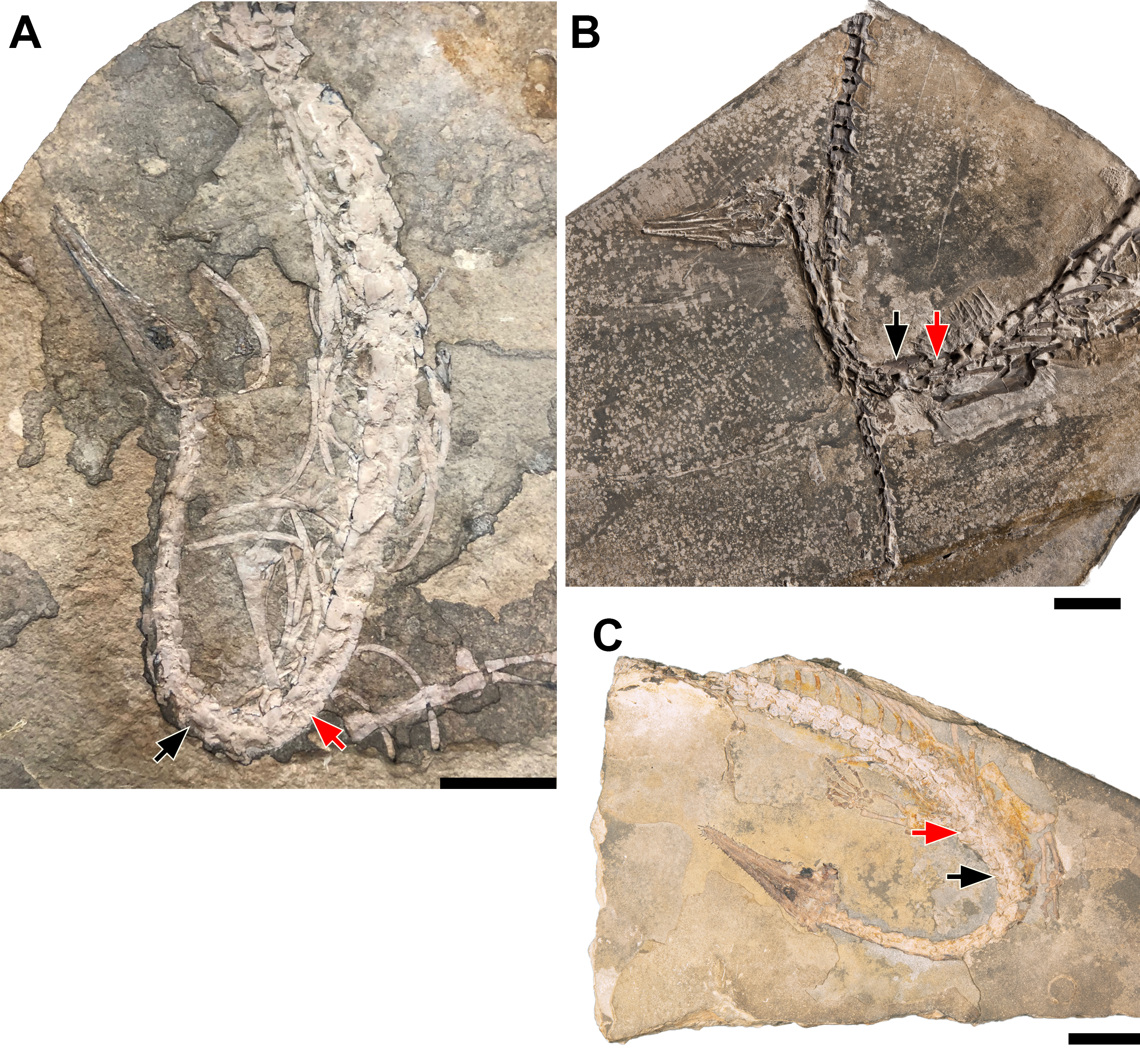

Supplement: Figure S2 — Black arrows point to the thirteenth and last cervical according to Piñeiro et al. (2021), red arrows point to the apparent beginning of the displaced ribcage. Scalebar 20 mm. A. “Brazilosaurus sanpauloensis” holotype NHMS 2187 described by Shikama & Ozaki (1966), photograph by Makoto Manabe (NHMS). B. PIMUZ A/III 513, photograph by Carola Radke (MB). C. ROM 28496, photograph by Antoine Verrière. [file peerj-10-13866-s003.png]
